# Retrospective assessment of clinical global impression of severity and change in GM1 gangliosidosis: a tool to score natural history data in rare disease cohorts

**DOI:** 10.1186/s13023-025-03614-6

**Published:** 2025-03-14

**Authors:** Connor J. Lewis, Jean M. Johnston, Silvia Zaragoza Domingo, Gilbert Vezina, Precilla D’Souza, William A. Gahl, David A. Adams, Cynthia J. Tifft, Maria T. Acosta

**Affiliations:** 1 Office of the Clinical Director and Medical Genetics Branch, National Human Genome Research Institute, National Institutes of Health, 10 Center Drive, Bethesda, MD USA; 2 Neuropsynchro, Barcelona, Spain; 3 Division of Diagnostic Imaging and Radiology, Children’S National Hospital, Washington DC, USA; 4 Medical Genetics Branch, National Human Genome Research Institute, 10 Center Drive, Bethesda, MD USA

**Keywords:** Rare diseases, Clinical trials, GM1 gangliosidosis, Lysosomal storage disorders, Clinical global impressions scale

## Abstract

**Background:**

Clinical trials for rare diseases pose unique challenges warranting alternative approaches in demonstrating treatment efficacy. Such trials face challenges including small patient populations, variable onset of symptoms and rate of disease progression, and ethical considerations, particularly in neurodegenerative diseases. In this study, we present the retrospective clinical global impression (RCGI) severity and change (RCGI-S/C) scale on 27 patients with GM1 gangliosidosis, a post hoc clinician-rated outcome measure to evaluate natural history study participants as historical controls for comparisons with treated patients in a clinical trial.

**Methods:**

We conducted a systematic chart review of 27 GM1 gangliosidosis natural history participants across 95 total visits. RCGI-S was assessed at the first visit and rated 1 (normal) to 7 (among the most extremely ill). Each subsequent follow-up was rated on the RCGI-C scale from 1 (very much improved) to 7 (very much worse). We demonstrate scoring guidelines of both scales with examples and justifications for this pilot in GM1 gangliosidosis natural history participants. The convergent validity of the RCGI scales was explored through correlations with magnetic resonance imaging (MRI) and the Vineland Adaptive Behavioral Scales.

**Results:**

We found strong association between the RCGI-S scores with gray matter volume (r(14) = −0.81; 95% CI [−0.93, −0.51], *p* < 0.001), and RCGI-C scores significantly correlated with increases in ventricular volume (χ^2^(1) = 18.6, *p* < 0.001). Baseline RCGI-S scores also strongly correlated with Vineland adaptive behavioral composite scores taken at the same visit (r(14) = −0.72; 95% CI [−0.93, −0.17], *p* = 0.02).

**Conclusion:**

RCGI-S/C scales, which use the clinical evaluation to assess the severity of disease of each patient visit over time, were consolidated into a single quantitative metric in this study. Longitudinal RCGI-C scores allowed us to quantify disease progression in our late-infantile and juvenile GM1 patients. We suggest that the retrospective CGI may be an important tool in evaluating historical data for comparison with changes in disease progression/mitigation following therapeutic interventions.

## Introduction

Rare diseases in the US are defined by a prevalence of fewer than 200,000 individuals [1, 2]. Although individually unusual, rare diseases cumulatively affect approximately 4% of the global population and represent millions of individuals [3, 4]. Clinical trials pose a particular challenge to the rare disease community, since they are often not amenable to a traditional double-blind placebo-controlled design [5]. Rare disease clinical trials must overcome small patient populations, varying patient ages and rates of disease progression, and inconsistent clinical trajectories [6]. In addition, progressive or degenerative diseases pose ethical challenges since they often cannot have a placebo control group, typically the gold standard for clinical trials [7]. Innovative solutions are urgently needed for clinical trial design for rare diseases.

GM1 gangliosidosis is an ultra-rare disease with an incidence of 1 in 100,000–200,000 births and an estimated national prevalence of 1,600–3,200 cases and global prevalence of 40,000–80,000 cases [8]. GM1 gangliosidosis is a recessive lysosomal storage disorder caused by biallelic mutations in *GLB1* leading to absent or decreased β-galactosidase enzyme activity responsible for the initial step in the degradation of complex gangliosides [9]. The lack of β-galactosidase activity results in the toxic accumulation of GM1 ganglioside, predominantly in the central nervous system (CNS) where its rate of synthesis is the highest [10, 11].

Although predominantly affecting the CNS, GM1 gangliosidosis is a multisystem disease also afflicting the musculoskeletal and cardiac systems [12, 13]. Atrophy of the cerebrum and cerebellum, with enlargement of the lateral ventricles and demyelination, are apparent on magnetic resonance imaging (MRI) [14, 15]. GM1 can be classified into three subtypes based on age of onset and rate of progression, with Type I infantile onset before 6 months of age and the most rapid progression; Type II (late-infantile) and Type II (juvenile) have symptom onset at 1–2 and 4–5 years, respectively. Type III patients have onset of symptoms in early adulthood and the slowest disease progression [8]. GM1 is uniformly fatal and has no approved therapies [16, 17].

Natural history studies, involving both empirical clinical evaluations and chart reviews, provide valuable information for clinical trial design. A paucity of researchers and clinicians follow cohorts of rare disease patients, acquiring knowledge through both empirical clinical evaluations and chart reviews. Longitudinally, researchers carefully phenotype patients to propose meaningful endpoints for disease-modifying trials and enhance supportive care [5]. A clear understanding of the clinical presentation, variability in symptom progression at different developmental ages, and appearance of lesser-known manifestations are important to monitor the effects of therapeutic interventions. Furthermore, natural history studies provide researchers with the opportunity to evaluate which clinical outcome assessments are fit-for-purpose for the concept of health and relevant to patients and their families to be used in subsequent trials in accordance with the Food and Drug Administrations’ Guidelines around Patient Focused Drug Development [18]. Additionally in degenerative diseases, natural history studies can be utilized as historical controls to assess therapeutic intervention [5].

The Clinical Global Impressions of Severity and Change (CGI-S/C) are clinician-based scoring systems extensively used in clinical care and clinical trials. They have become gold standards for quantifying and tracking patient progress and treatment response over time [19, 20] and for validating new outcome measurements for conditions affecting the skin, nervous system, musculoskeletal system, and behavior [21]. The flexibility of the CGI scales permits clinical expertise to be leveraged to establish the most critical factors of a clinical assessment [22].

In this study, we present a retrospective analysis of GM1 patients’ clinical severities using Clinical Global Impression of Severity and Change (RCGI-S/C) scales to communicate the clinician’s view of patient morbidity and disease progression, using blended (qualitative and quantitative) information collected from each patient´s clinical record. Data from a type II GM1 Gangliosidosis cohort were analyzed and scored to determine the severity at the first visit and the trajectory of disease progression up to the last visit available. Here, we present the description of the scale and the first application to a cohort of Type II GM1 gangliosidosis patients.

## Methods

### Patients

Children and adults between the ages of 1 and 31 years with a confirmed diagnosis of GM1 gangliosidosis, both by β-galactosidase enzyme analysis and molecular analysis showing biallelic mutations in *GLB1*, were enrolled in clinical protocol “Natural History of Glycosphingolipid & Glycoprotein Storage Disorders” (NCT00029965) and they or their families gave written, informed consent. Symptom onset was between 11 months and 6 years. Participants were seen for one-week evaluations at the National Institutes of Health Clinical Center at intervals of 1–2 years between 2010 and 2020 [17]. They underwent a standard and systematic battery of testing during each visit; the cohort’s clinical data were previously described [17].

### RCGI-S/C scales

Based on the standard CGI scoring [19, 20], we designed a specific RCGI-S/C scale for GM1 gangliosidosis to retrospectively assess this cohort. Each patient’s clinical severity was scored based upon a consensus reached among three researchers (JMJ, CJT, MTA), using their clinical experience and a targeted literature review about Type II GM1 gangliosidosis and its related comorbidities. The CGI sub scores or subscales included retrospective data on *Developmental evaluations*, *seizure presence* and *presentation, presence of aspirations*, *gastrointestinal evaluation*, *skeletal survey*, *liver enzyme tests*, and *sleep disturbances* (Fig. 2). The scoring process included: (i) Collection and organization of the information included in the medical records made (including information from patients and relatives) by the site coordinator research nurse; and (ii) Review of the available information by three members of the staff, two of whom had extensive contact with the patient cohort over the 10-year duration of the natural history study (JMJ and CJT) and a third experienced pediatric neurologist not involved in the study.

Scoring instructions for RCGI-S included all clinical aspects, from number and severity of comorbidities to the clinical impact on the family and patient’s life, encompassed by one single score. A patient with multiple mild comorbidities (mild developmental delay, speech problems, fine and gross motor difficulties, sleep abnormalities behavioral problems) may score lower on the severity scale compared to patients with fewer but more severe comorbidities (severe and difficult to control seizure disorder, swallowing and deglutition problems, and severe developmental delays). The final score for baseline severity and change at each time point represented a verbal consensus among the three researchers resulting from an open discussion.

**Fig. 1:**
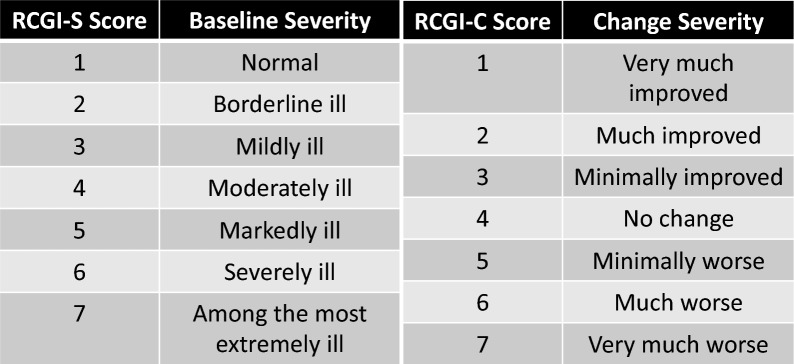
Retrospective Cognitive Global Impression (RCGI) Scales scoring, adapted from Busner and Targam (2007) [19, 20]. Baseline and first evaluation were scored with the RCGI severity (RCGI-S) scale (left). Follow-up evaluations were scored with the RCGI change scale (right) as a comparison between the baseline evaluation and each subsequent follow-up

**Fig. 2:**
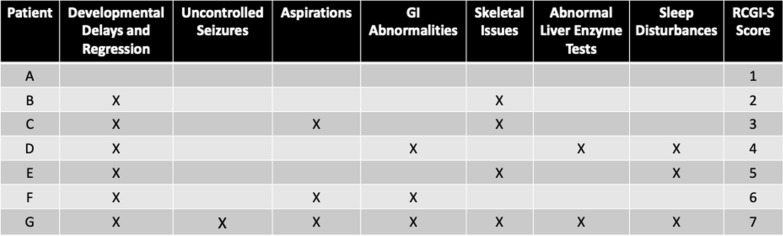
Summary of Notable Comorbidities and Corresponding RCGI-S Scores by Patients Included in Baseline Descriptions. An ‘X’ represents the presence of the comorbidity, and ‘N/A’ indicates that the assessment was not completed at the baseline visit

The first visit in the medical record from the registry cohort was considered as the baseline evaluation of severity, where RCGI-S scale scores ranged from 1 (Normal) to 7 (Among the most extremely ill subjects) (Fig. 1). Clinical severity evaluated for each subsequent visit in the medical record was assessed as the change compared to the baseline score as RCGI-C ranging from 1 (Very much improved) to 7 (Very much worse) (Fig. 1). Scores were correlated with already reported metrics of disease severity in GM1 patients, i.e., neuroimaging and neurodevelopmental scales [23–25] (Fig. 2).

### Neuroimaging

Since atrophy of the cerebrum, white matter, and enlargement of the ventricles have been associated with GM1 progression [23–25], volumetric analysis of T1-weighted MRI was performed post hoc. Images were acquired on a 3 T Phillips Achieva system with a 3D magnetization-prepared rapid acquisition gradient echo (MPRAGE) sequence and a slice thickness of 1 mm. T1 weighted images were sent through Freesurfer’s (v7.4.1) *recon-all* pipeline where motion correction, intensity normalization, and segmentation were performed to calculate total gray matter, white matter, and ventricular volume [26–34].

### Neurodevelopment

For behavioral evaluations, the Vineland Adaptive Behavior Scales [35, 36] is a semi-structured clinical interview conducted with the caregiver. In previous work with these patients, the Vineland scores were significantly impaired relative to typically developing peers [17, 35, 36]. Here, we employed the Adaptive Behavior Composite (ABC), a validated overall metric of functional impairment. The ABC is a standard score with a population mean of 100 and SD of 15. Due to the length of this study, different formats of the Vineland were used (II and 3); the Vineland-II was used prior to 2016, and the Vineland-3 was used from 2016 on since its release [35, 36]. ABC standard scores were used from both editions, since they both have a population mean of 100 and SD of 15 [35, 36]. Ten patients (8 juvenile and 2 late-infantile) had corresponding baseline RCGI-S scores with either a corresponding Vineland-3 or Vineland-II score. Vineland-3 scores were used when available (n = 4), otherwise Vineland-II (n = 6) scores were used if the participant had no Vineland-3 evaluation at the baseline visit.

### Statistics

Statistical analyses were performed in R (v4.3.1) [37] where linear and multivariate regression models were used to evaluate the effect of patient age and GM1 gangliosidosis subtype on baseline RCGI-S scores. Late-infantile GM1 gangliosidosis patients were assigned a value of 0 and juvenile patients were assigned a value of 1 to evaluate the effect of GM1 gangliosidosis subtype. Linear mixed effects modeling was created using the LME4 package [38, 39] and were used to evaluate the relationship between RCGI-C scores with GM1 subtype, participant age, and the elapsed time since the baseline evaluation. A subject-level random intercept was used to account for repeated measures [39, 40]. Pearson correlations were also calculated in R between cross-sectional volumetric MRI data (gray matter, white matter, and ventricle volume) and baseline RCGI-S scores (considered as continuous measures). Pearson correlations were also calculated between RCGI-S scores and Vineland Adaptive Behavioral composite standard scores available at baseline evaluation [17, 35, 36]. *P*-values were calculated from the Pearson Product-Moment correlation using the *cor.test* function in R [37]. Linear mixed effects modeling was also used to evaluate the percent change in volumetric MRI data (fixed effect) with RCGI-C scores. A subject-level random intercept was used to account for repeated measures [38–40]. Of the 10 patients with Vineland data, there were 16 longitudinal time points with matching RCGI-C and corresponding Vineland-3 or Vineland-II scores. A percentage change in Vineland scores was used to account for relative changes at the lower portions of the scale.

## Results

For this pilot study, the study sample included the records of 9 late-infantile age 5.4 ± 1.7 years and 18 juvenile GM1 patients aged 11.5 ± 4.7 years. There were 95 total visits (late-infantile: 22, juvenile: 73) over a period of 3.7 ± 4.1 years for late-infantile patients and 5.3 ± 4.0 years for juvenile patients. The average follow-up interval between evaluations was 2.6 ± 2.3 years for late-infantile patients and 1.7 ± 1.3 years for juvenile patients.

### RCGI-S cases with scores 1–7

#### *Patient A, RCGI-S* = *1 (normal)*

Patient A, a 16-month-old male with a diagnosis of juvenile GM1 was asymptomatic at the time of first evaluation, had normal development, and no GM1-associated comorbidities. He was diagnosed based on the diagnosis of an older sibling who was symptomatic by four and a half years.

#### *Patient B, RCGI-S* = *2 (borderline Ill)*

Patient B, a nine-year-old male with juvenile GM1 presented with gait alterations and pain while walking. He had no sleep disturbances or feeding issues. He had decreased hip range of motion, osteopenia, and pectus carinatum. Liver enzymes were normal. Developmental assessment showed mild language delay, mild gait disturbance, and mild to moderate cognitive impairment. Initial symptoms included skeletal and bone-related abnormalities, neurological deficits, and developmental delays. Signs and symptoms of disease were minor and not specific for GM1. He was ambulatory and did not manifest a movement disorder, dystonia, sleep problems, or seizures.

#### *Patient C, RCGI-S* = *3 (mildly Ill)*

Patient C, a ten-year-old female with juvenile GM1 presented with language and motor developmental delays beginning at 5 years of age. GM1 was diagnosed at age 8. She had osteopenia and vertebral body deformities. A speech and swallowing study showed mild oral dysphagia without aspiration, oral motor sensory deficits, and moderate dysarthria. The family acknowledged some swallowing difficulties without choking. At her first evaluation, Patient C had motor and language impairments, skeletal abnormalities, and swallowing difficulties. She did not have seizures, aspiration, liver enzyme abnormalities, or severe behavioral, developmental or cognitive delays.

#### *Patient D, RCGI-S* = *4 (moderately Ill)*

Patient D, a nine-year-old male with late-infantile GM1 presented with developmental regression and mild intellectual disability. He also had anxiety, mood dysregulation, short attention span, frequent falls, significant fine and gross motor deficits, sleep disturbances, vomiting, and feeding problems resulting in a recent ten-pound weight loss. Abdominal ultrasound showed splenomegaly and mild hepatomegaly. Comorbidities included developmental regression, gastrointestinal and feeding problems, intellectual disability, behavioral difficulties, and sleep disturbances.

#### *Patient E, RCGI-S* = *5 (markedly Ill)*

Patient E, a twelve-year-old female with juvenile GM1, presented with severe developmental delay, motor and cognitive regression, behavioral abnormalities, slurred speech, sleep disturbances, strabismus, and skeletal abnormalities including scoliosis, kyphosis, osteopenia, and a history of surgery for hip dysplasia and platyspondyly of the vertebral bodies. She had an abnormal abdominal ultrasound with hepatic parenchymal echotexture compatible with diffuse fibrosis or fatty infiltration. The liver enzyme and cerebrospinal fluid results were within the normal limits. Patient E did not develop other severe symptoms like seizures, pneumonia, or aspirations. However, her multiple and moderately severe comorbidities and disease-related surgeries led to a “markedly ill” score.

#### *Patient F, RCGI-S* = *6 (severely Ill)*

Patient F, an eight-and-a-half-year-old male with late-infantile GM1, presented with focal and generalized seizures and a history of early developmental regression followed by severe cognitive and motor impairments. He had a sleep disturbance without apnea, gastrostomy tube (G-tube) placement due to aspiration pneumonia, and cortical blindness. He was totally dependent for care and minimally interactive with his environment. Multiple bony abnormalities included osteoporosis; splenomegaly was noted This constellation of severe comorbidities yielded a score of severely ill.

#### *Patient G, RCGI-S* = *7 (among the most extremely Ill)*

Patient G, a 4-and-a-half-year-old female with late-infantile GM1, presented with severe developmental delays and regression, daily uncontrolled seizures, sleep apnea, aspiration pneumonia, G-tube dependency, abnormal hepatic enzymes (e.g., GGT (126 U/L), and multiple bone abnormalities including kyphoscoliosis, dislocated left hip, deformed femoral epiphysis, and osteopenia. These debilitating comorbidities warranted a score of most extremely ill.

### RCGI-C cases

Two examples illustrate the scoring of clinical change with more than 10 years of follow up.

#### *Patient H (RCGI-S* = *4 “moderately Ill”)—baseline*

Patient H was an 18-year-old male diagnosed with juvenile GM1 at 11 years of age. Concerns for developmental delays and regression were present at 5 years of age. By age 9 he had ataxia, difficulty walking, stuttering, and an abnormal EEG without seizures. At age 11, he had bilateral corneal clouding, which raised the question of a potential lysosomal storage disease. At his baseline visit, he presented with cognitive and motor impairments with minimal ability to get out of bed and stand up. There was muscle atrophy with lower extremity contractures, generalized spasticity, limited range of motion, and bone abnormalities, including bilateral hip dysplasia, significant osteopenia, pectus excavatum, and kyphosis. CSF results and liver enzymes were unremarkable. He had oral motor apraxia and dysarthria but a normal swallowing study. He was receiving Botox injections in lower extremities and medications for sleep, seizures, attention deficits, behavioral problems, and constipation.

#### *Follow up #1 (22 years old), RCGI-C* = *4 (no change)*

Patient H’s only complaint at this visit was worsening constipation, not considered a significant change.

#### *Follow up #2 (23 years old), RCGI-C* = *4 (no change)*

Patient H’s family reported mild improvement in his mood and behavior. He had sinus infections during the past year and his liver enzyme tests remained normal.

#### *Follow up #3 (24 years old), RCGI-C* = *4 (no change)*

Patient H’s speech and swallow test were unchanged, and liver enzyme tests and ECG were both normal.

#### *Follow up #4 (25 years old), RCGI-C* = *5 (minimally worse)*

Patient H’s family reported that he was pain free with some improvement in upright mobility, but his cognitive function was worse, and he developed obsessive compulsive-disorder-like symptoms. His speech deteriorated and he developed progressive dysarthria with increased stuttering. His speech and swallowing testing showed no changes; liver enzymes and ECG were normal.

#### *Follow up #5 (27 years old), RCGI-C* = *5 (minimally worse)*

Since the last evaluation, Patient H had one seizure-like episode and had bone pain. He retained the occasional ability to stand up and take a few steps with support. Liver enzyme tests, CSF, and swallow tests remained normal. Language comprehension was preserved despite impaired expressive language. He was scored as “minimally worse” given the seizure like event and the pain.

#### *Follow up #6 (29 years old), RCGI-C* = *6 (much worse)*

Two years later, Patient H’s parents stated he had deteriorated, with worsening cognitive and behavioral deficits, swallowing difficulties, and chocking with liquids. His level of activity decreased, and he had developed visual impairment, urinary incontinence and retention along with severe constipation. The abdominal ultrasound was unremarkable, and the ECHO showed no noticeable changes from follow-up #3.

#### *Follow up #7 (31 years old), RCGI-C* = *6 (much worse)*

This evaluation occurred around 2 years after the previous one. He had lost his ability to stand and walk. His speech and ability to communicate and interact with family members and surrounding environment had deteriorated. He had poor feeding due to problems chewing and swallowing food. He lost movement in his left shoulder but was without pain. The ECHO showed mild tricuspid, mitral, and pulmonic regurgitation, with aortic dilation. His sleep efficiency was 60% (335 min out of 558), with hypopnea and apnea. Patient H had no episodes of pneumonia but had reduced lung volume with regions of atelectasis. His dual-energy X-ray absorptiometry (DEXA) showed significantly reduced bone mass and contractures of the knees and ankles. He had a stable EEG, liver enzymes, and remained seizure-free.

#### *Patient I (RCGI-S* = *4 “moderately Ill”)—baseline*

Patient I, a 4.5-year-old female, was diagnosed with late-infantile GM1 at age 4. She had developmental delay affecting fine and gross motor skills as well as cognition. Her problems started at the age of 2. At her first evaluation, she could crawl but required a gait trainer to walk; she also required some assistance feeding. She had seizures beginning at age 3, well controlled on her current treatment regimen.

#### *Follow up #1 (7 years old), RCGI-C* = *5 (minimally worse)*

Since the last visit, she developed insomnia and daytime sleepiness and was receiving melatonin. She had no seizures since the last visit but had worsening cognitive function and constipation. Her mobility had been reduced to scooting. Her speech and swallowing study showed signs of silent aspirations; this was corrected with her thoracic vest. When compared to baseline, patient I had developed sleep disturbances, further cognitive impairments, reduced mobility, and constipation, demonstrated disease progression.

#### *Follow up #2 (8 years old), RCGI-C* = *6 (much worse)*

Patient I’s parents stated that her sleep disturbances had become worse, and she now required clonidine and melatonin. She had a drastic regression of skills, including fine and gross motor along with weight loss and a hospitalization for pneumonia. The patient consistently bit her fingers requiring the removal of teeth. She was irritable and non-interactive. She had worsening constipation. Her seizures remained well-controlled. She had developed hip dysplasia, neck spasms, and worsening aspiration risks on a speech and swallow study. She lost her independent floor mobility. The significant weight loss, finger biting, irritability, and mobility impairments led to the worsening score.

#### *Follow up #3 (10 years old) RCGI-C* = *7 (very much worse)*

Since the previous visit, patient I required a G-tube placement for feeding and prevention of aspiration. She had recurrent pneumonias and seizure-like symptoms including tremors and abnormal eye movements. She had notable fine and gross motor losses when compared to her previous visit and had worsening cognitive function. In composite, her deterioration warranted a score of very much worse.

#### *Follow up #4 (18 years old) RCGI-C* = *7 (very much worse)*

Patient I was admitted to an intensive care unit with severe bradycardia and hypothermia requiring bilevel positive airway pressure (BIPAP). Her EEG showed evidence of cerebral dysfunction and epileptiform activity; her recent seizures were uncontrolled with medication.

### Correlation of RCGI-S/C scores with age, study duration, and GM1 subtype

**Fig. 3:**
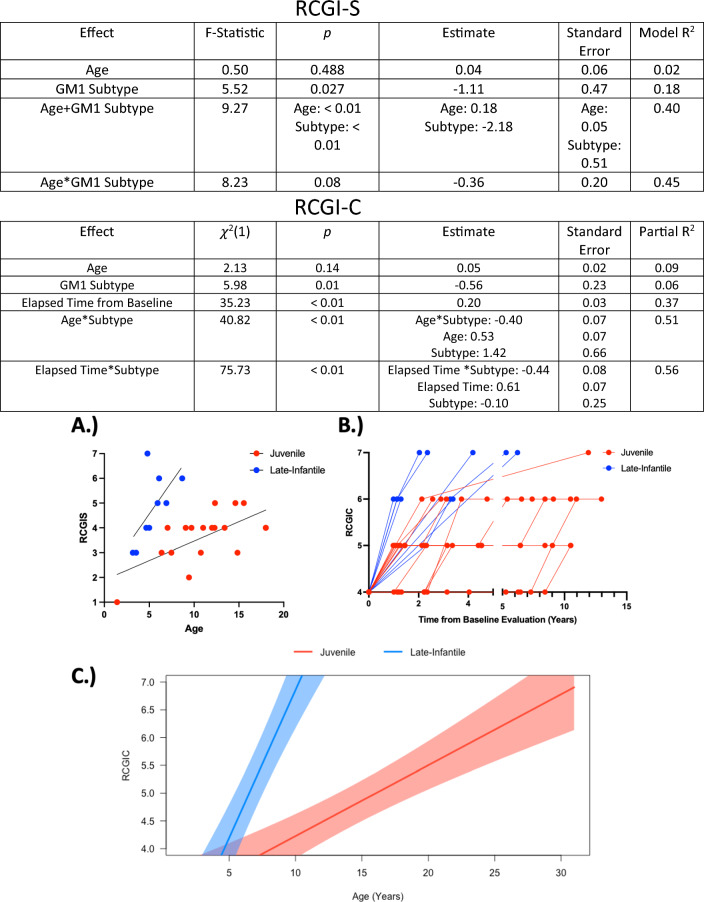
The effect of age and disease subtype on RCGI Scores. The linear and multivariate regression model outputs of the relationship between RCGI-S with patient age and GM1 gangliosidosis patient age. The linear mixed effects model outputs of the relationship between longitudinal RCGI-C with patient age, GM1 subtype, and time elapsed from baseline evaluation (years). **A** The relationship between participant age at the baseline evaluation and baseline RCGI-S score when disease subtype was accounted for. **B** The relationship between the time elapsed since the baseline evaluation and longitudinal RCGI-C scores with late-infantile GM1 patients shown in blue and juvenile GM1 patients shown in red. **C** Linear mixed effects model output demonstrating the relationship of longitudinal RCGI-C with GM1 disease subtype and participant age. The shaded region represents the 95% confidence interval

GM1 gangliosidosis subtype (*p* = 0.027) significantly influenced RCGI-S scores where juvenile patients had a 1.11 lower RCGI-S score when disease subtype was evaluated alone (Fig. 3). Participant age did not influence RCGI-S scores (*p* = 0.488) when evaluated alone. When participant age and GM1 subtype were evaluated together, juvenile patients had a 2.18 lower RCGI-S scores (*p* < 0.01) compared to late-infantile patients of the same age. Age also correlated with RCGI-S scores (*p* < 0.01) and increased by 0.18 for every year older the patient was at the baseline evaluation. The interaction between GM1 subtype and participant age did not influence (*p* = 0.08) baseline CGI-S scores.

The linear mixed effects modeling of RCGI-C scores showed participant age did not influence RCGI-C scores (*p* = 0.14) when evaluated alone. GM1 gangliosidosis subtype significantly influenced RCGI-C scores (*p* = 0.01) where juvenile GM1 gangliosidosis patients had a 0.56 lower RCGI-C score across all visits. The time (in years) elapsed between the initial evaluation and the evaluation being scored as comparison also influenced RCGI-C (*p* < 0.01), where on average GM1 patients had a 0.2 RCGI-C score increase per year. The interaction between the time elapsed since the baseline evaluation and GM1 gangliosidosis subtype significantly influenced RCGI-C scores (*p* < 0.01, Fig. 3B). Late-infantile patients had a 0.61 increase in RCGI-C score per year in the study, and juvenile patients had a 0.17 increase in RCGI-C score per year in the study. The interaction between GM1 gangliosidosis subtype and patient age also significantly influenced RCGI-C scores (*p* < 0.01, Fig. 3C). Late-infantile patients had a 0.53 increase in RCGI-C score per year, and juvenile patients had a 0.13 increase in RCGI-C score per year.

### Correlation of RCGI-S/C scores with neuroimaging and Vineland results

**Fig. 4:**
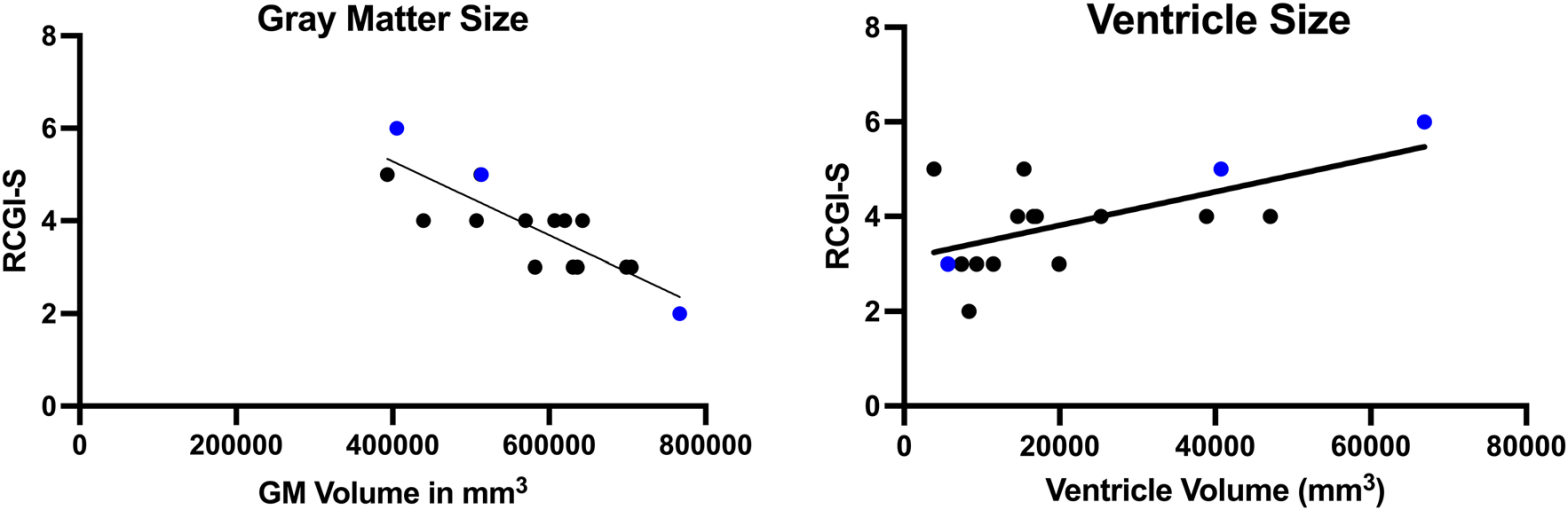
Correlations of MRI volume metrics with baseline RCGI-S. 16 patients had corresponding T1-weighted MRI scans at the time of first visit and RCGI-S evaluation. Late-infantile patients (n = 3) are shown in blue and juvenile patients (n = 13) are shown in black. A.) Gray matter volume showed the expected negative trend with baseline severity (r(14) = −0.81, 95% CI [−0.93, −0.51], *p* < 0.001). B.) Ventricle volume showed the expected positive trend with baseline severity (r(14) = 0.61, 95% CI [0.17, 0.85], *p* = 0.01)

Gray matter volume (r(14) = −0.81; 95% CI [−0.93, −0.51], *p* < 0.001) and ventricle volume (r(14) = 0.61; 95% CI [0.17, 0.85], *p* = 0.01) showed negative and positive trends with severity at baseline (RCGI-S scores), respectively (Fig. 4), consistent with the disease progression of both biomarkers. In contrast, white matter volume (r(14) = 0.36; 95% CI [−0.19, 0.73], *p* = 0.18) was not found to correlate with baseline RCGI-S scores. For the disease progression evaluations (RCGI-C), linear mixed effects modeling showed that all, i.e. the percent change in gray matter (χ^2^(1) = 6.1, *p* = 0.01), ventricle volume (χ^2^(1) = 18.6, *p* < 0.001), white matter volume (χ^2^(1) = 5.3, *p* = 0.02), and total brain volume (χ^2^(1) = 12.5, *p* < 0.001) correlated significantly with longitudinal RCGI-C scores at all evaluations time points. Figure 6 demonstrates the positive relationship of RCGI-S scores with global brain atrophy between patients. Figure 7. demonstrates the longitudinal stability of Patient H in terms of neuroimaging, which was similarly reflected in the RCGI-C score.

**Fig. 5:**
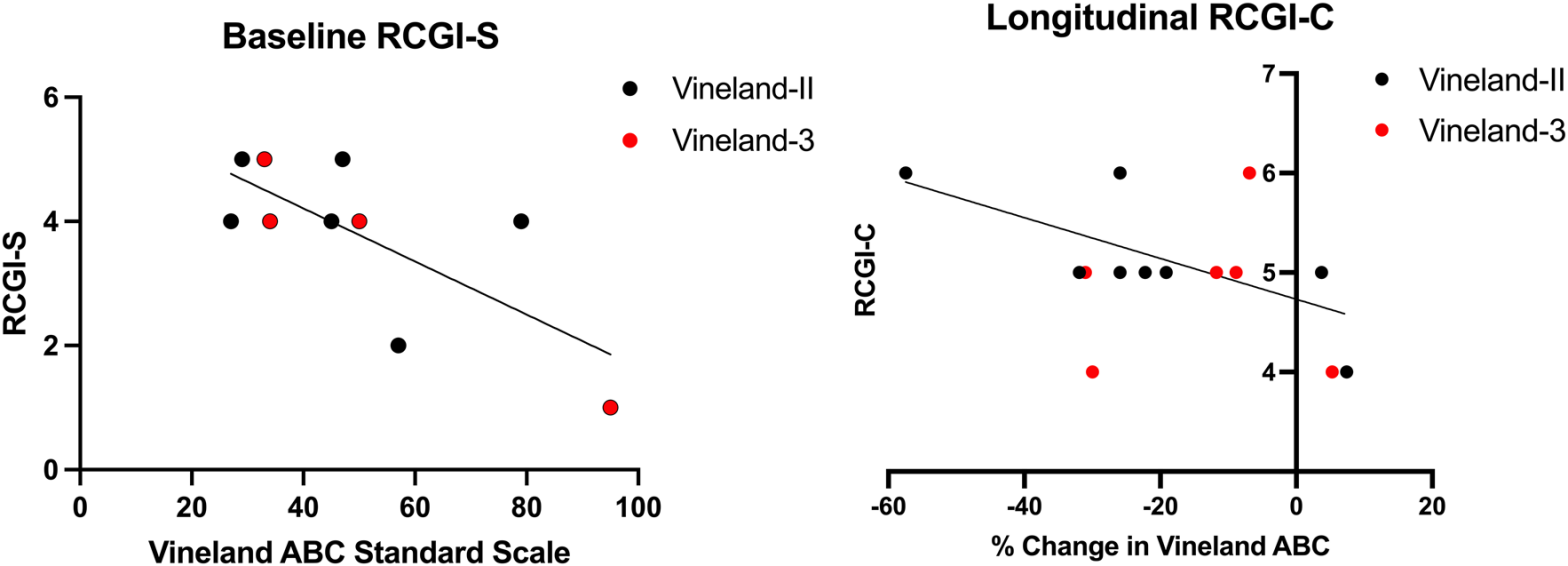
Correlations of Vineland scales with baseline RCGI-S. 10 patients had corresponding Vineland scores at the time of first visit and RCGI-S evaluation. A.) Vineland scores showed the expected negative trend with baseline severity (r(14) = −0.72, 95% CI [−0.93, −0.17], *p* = 0.02). B.) Of those 10 patients, 16 time points had corresponding Vineland (either Vineland II or 3) scores and RCGI-C scores. Percent change in Vineland scores did not correlate with RCGI-C (r(8) = −0.48, 95% CI [−0.79, 0.02], *p* = 0.06)

For behavioral evaluations, the Vineland adaptive behavioral composite standard scores showed a statistically significant negative relationship with baseline severity RCGI-S (r(14) = −0.72; 95% CI [−0.93, −0.17], *p* = 0.02, Fig. 5). However, percent change in Vineland scores did not correlate with RCGI-C (r(8) = −0.48; 95% CI [−0.79, 0.02], *p* = 0.06). In summary, Vineland scores and MRI Volumetric analysis of the ventricles and gray matter volume both correlated significantly with baseline severity according to the RCGI-S. For the change in severity (RCGI-C), the MRI volumetric analyses, including changes in total brain volume, gray matter volume, white matter volume, and ventricle volume, all correlated with RCGI-C.

## Discussion

The challenges involved in rare disease clinical trials relate to sample size, a control population, randomization, and variability in disease severity, age of onset, and rate of disease progression. Researchers have turned to natural history studies to assess disease progression following therapeutic intervention, using historical controls. In this study, we demonstrated the use of retrospective CGI-S/C scales to provide a clinician-rated severity and change scale to assess clinical data in a rare neurodegenerative disease, i.e., GM1 gangliosidosis.

**Fig. 6:**
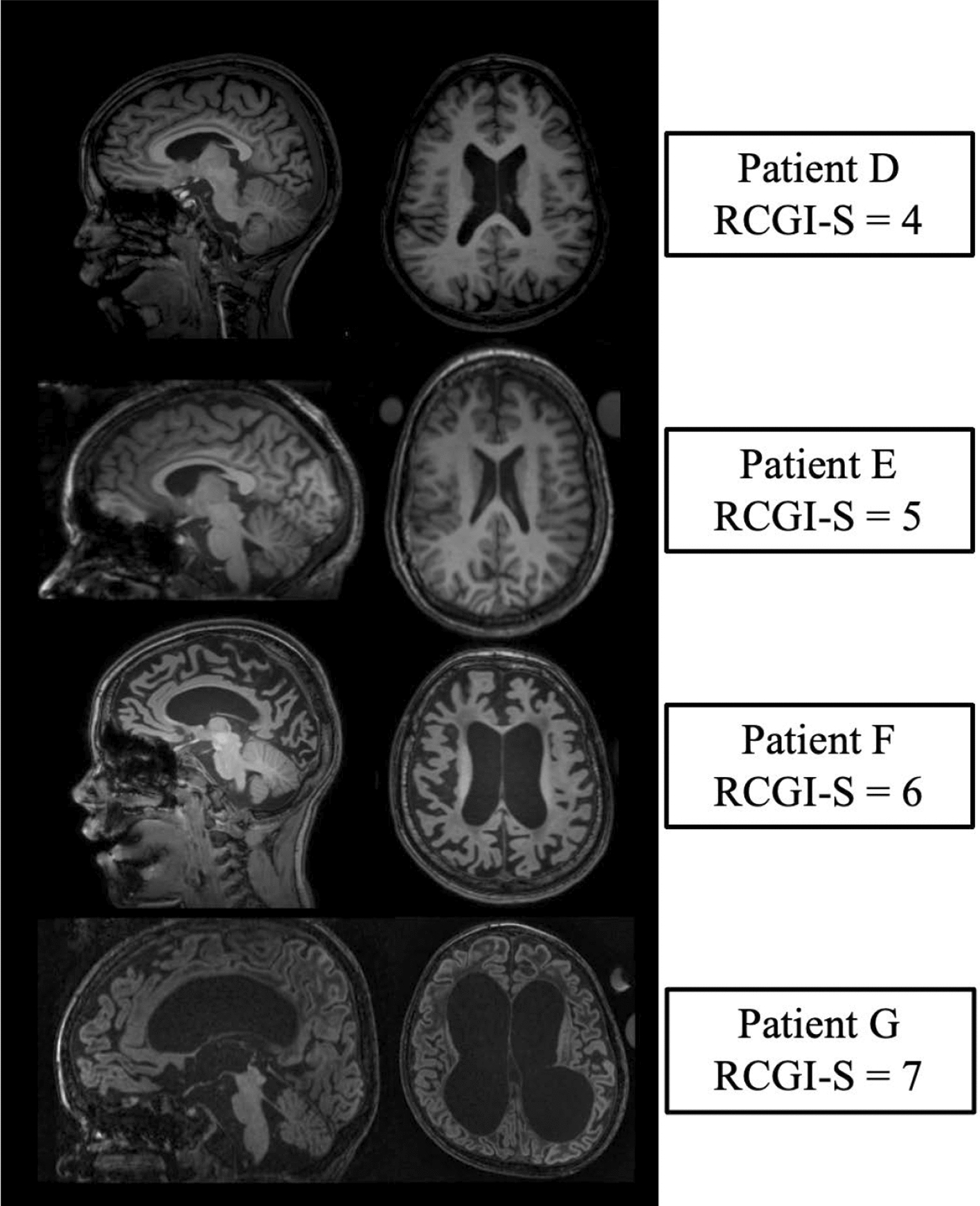
Baseline magnetic resonance imaging for patients D, E, F, and G. Demonstrating worsening atrophy of the corpus callosum and cerebrum, and ventricular enlargement with worsening RCGI-S score

In this example, RCGI-S scores ranged from 1(Normal) to 7(among the most extremely ill) spanning the RCGI-S scale. As expected, the RCGI-S increased consistently with the patients’ comorbidities in terms of prevalence and severity (Fig. 2). RCGI-S scores were also higher in late-infantile GM1 patients when compared to juvenile GM1 patients of the same age (Fig. 3A), which was an expected result due to the separate clinical trajectories of the two sub-types [17]. Similarly, both baseline gray matter volume and ventricle volume significantly correlated with the baseline RCGI-S; reductions in cerebral volume and subsequent enlargement of the ventricles corresponded to a more severe baseline clinical presentation (Figs. 4 and 6). Finally, we found RCGI-S correlated with Vineland Adaptive Behavioral Composite Scores, further validating the scale (Fig. 5).

**Fig. 7:**
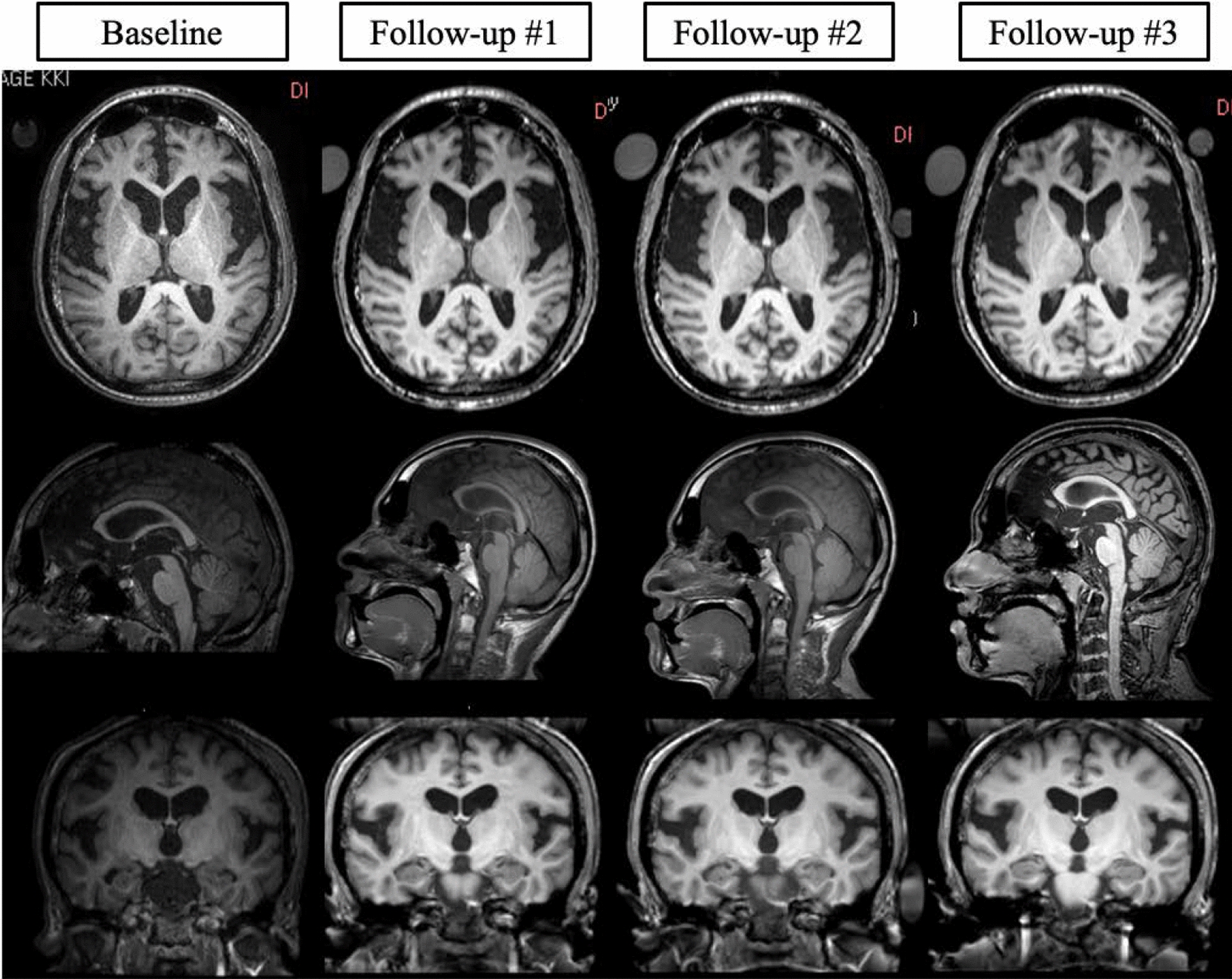
Longitudinal magnetic resonance imaging of patient H. Highlighting baseline cerebrum atrophy and longitudinal stability. Patient H was rated a CGI-C = 4 (No Change) at all three follow-up visits when compared to baseline

RCGI-C scores ranged from 4 (No change) to 7 (very much worse) in our GM1 cohort. We found that the appearance of new disease-related comorbidities, and any increase in the severity of these morbidities, corresponded with a worsening RCGI-C score. RCGI-C scores also increased faster (0.4 per year) in late-infantile patients over the same follow-up duration when compared to juvenile patients (Fig. 3B and C). Also, changes in white matter, gray matter, total brain size, and ventricle volume correlated with the RCGI-C score; indeed, longitudinal stability in CGI-C corresponded with biomarker MRI stability over six years in one of the studied cases (Fig. 7). Since the increasing rate of the score accurately reflects the rate of disease progression as confirmed by imaging biomarkers, the RCGI scales can inform prospective clinical data collection for clinical trials and ongoing natural history studies.

CGI scales are limited by their subjective nature. However, with adequate methodological settings, a correlation with more objective measures, and expert clinicians involved, these scales may be accurate and robust in showing clinical severity and progression. In rare diseases, biomarkers and clinical measurements are not always well defined or refined at the beginning of natural history data collection [6, 40]. Disease manifestations can be varied or absent. For example, seizures are a hallmark of GM1 but are extremely varied in their clinical presentation and can be absent throughout much of the course of the disease [17]. Some patients with severe GM1 have uncontrolled seizures multiple times per day (Patient G) while others never develop them (Patient H). Global impression measures can provide a pragmatical solution to harmonize clinical criteria useful for clinical research. In fact, the RCGI-S/C proved useful to inform and power early phase for our GM1 Intravenous Gene therapy Trial (NCT03952637).

Another limitation of the RCGI-S/C scales was highlighted in our GM1 analysis. The natural progression of GM1 showed only a worsening prognosis, with RCGI-C scores between 4 and 7 (Fig. 3B). This was expected due to the degenerative nature of GM1 but nevertheless represents a narrow band of scores between “normal” or “no change” and “among the most extremely ill” or “very much worse” for the RCGI-S and RCGI-C scales, respectively; only 3 scores are available for evaluating potential benefits resulting from therapeutic intervention. Furthermore, the nomenclature of the traditional CGI-I scale assumes clinical improvements in patients; given the degenerative nature of GM1, this may be inappropriate.

Validation is the next step in establishing inter-rater agreement, not covered in this first version of the instrument. One rater or group of raters should score all patients at all timepoints with the potential involvement of a separate second rater (or group of raters) to create a centralized rating. To further control for intra-rater reliability, the entire scoring process of the retrospective data should be completed within a specified time interval [41]. Furthermore, for future studies in our research unit we are considering including the Patient/Careers Global Impression (P/CGI) scales to capture important feedback from families to assess the burden and severity. This will help anchor the CGI scores to be in line with patient-focused drug development guidance [42].

This study, aiming to pilot the novel instrument, was also limited by a small sample size (n = 27) and variable follow-up intervals for patients due to the real-world nature of the cohort. Although several statistical methods could be used to infer the progression in non-available timelines, e.g., using slope rates as measures/endpoints instead of point-measures, we would like to further demonstrate the efficacy of the retrospective scale, Hence, replicate analysis of a cohort with a larger sample size and more consistent evaluations will be conducted by applying the lessons learned to a wider sample of the registry cohort. However, recruitment of a large cohort poses an immense challenge for a rare disease like GM1 [43]. Future investigations should implement blinded raters for RCGI scoring to limit hierarchical influence and to confirm the validity of ratings when only the information collected in medical records is considered.

## Conclusion

In this study we used the CGI-S and CGI-I to evaluate retrospective data from a rare neurodegenerative disease, GM1 gangliosidosis. Experienced clinicians utilized clinical signs and symptoms in GM1 patients to create a centralized baseline score of disease severity and subsequent scores for disease progression, I.E., CGI-S and CGI-C. The measures correlated with more objective measures of disease including brain atrophy and adaptive behavior. We suggest that retrospective CGI scoring can be a clinically meaningful addition to outcome measures in the context of a clinical trial, particularly in a rare disease with clinical variability when sufficient natural history information and clinical expertise are available.

## Data Availability

The data described in this manuscript are available from the corresponding author upon reasonable request.
